# Taxonomic revision of the Neotropical genus *Rhabdotylus* Lutz, 1913 (Diptera: Tabanidae)

**DOI:** 10.3897/BDJ.7.e36277

**Published:** 2019-07-10

**Authors:** Mauren Turcatel

**Affiliations:** 1 National Museum of Natural History, Smithsonian Institution, Washington, D.C., United States of America National Museum of Natural History, Smithsonian Institution Washington, D.C. United States of America

**Keywords:** Horse fly, identification key, Tabanidae, *
Rhabdotylus
*, taxonomy

## Abstract

**Background:**

Here I revise the Neotropical genus *Rhabdotylus* Lutz, 1913 (Tabaninae: Diachlorini), including redescription of three species that range from Guatemala to Argentina: *Rhabdotylus rubrum* (Thunberg, 1827), *Rhabdotylus venenatum* (Osten Sacken, 1886), and *Rhabdotylus viridiventris* (Macquart, 1838).

**New information:**

*Rhabdotylus planiventris* (Wiedemann, 1828) is established as a junior synonym of *R. rubrum*, syn. nov. A dichotomous identification key based on external morphological characters is provided.

## Introduction

*Rhabdotylus* Lutz, 1913 (Diptera: Tabanidae: Diachlorini) is a Neotropical genus, and it was described based on *Tabanus planiventris* Wiedemann, 1828. In 1925, Enderlein treated *Rhabdotylus* and *Dicladocera* Lutz, 1913 as synonyms; however, in the same publication, he designated *T. planiventris* as the type species of his new genus *Gymnochela* (Enderlein 1925). Kröber, in 1932, redescribed *T. venenatum* Osten Sacken, 1886 (as *venenatus*, error) and listed *T. viridiventris* Macquart, 1838 to Gymnochela (subgenus Amphichlorops) (Kröber 1932); in 1934, he treated both *Rhabdotylus* and *Gymnochela* as synonyms of *Amphichlorops* Lutz, 1913 (Kröber 1934). Fairchild, noting the similarities in the head morphology of *Rhabdotylus* and *Stibasoma* Schiner, 1867, proposed *Rhabdotylus* as a subgenus of *Stibasoma* (Fairchild 1942). Carrera and Lane acknowledged that *Rhabdotylus* should be reinstated as a genus, and redescribed *T. viridiventris* as *Rhabdotylus* (Carrera and Lane 1945). In 1967, Philip stated that *T. rubrum* Thunberg, 1827 (as *ruber*, error) “should be listed as a separate species of *Stibasoma*” (Philip 1967: 1236). Later, Trojan revalidated *Rhabdotylus* as a genus, based on the differences in body pilosity and leg structure, and stated that three species were known; however, only *R. planiventris* (as *planiventre*, error) and *R. venenatum* (as *venatum*, error) were named in his examined material list (Trojan 1998). Turcatel et al. revised the genus *Stibasoma* and, following the classification proposed by Trojan, treated *Rhabdotylus* as a separate genus (Turcatel et al. 2010). The most recent catalogue of Neotropical Tabanidae (Coscarón and Papavero 2009) lists four species under *Rhabdotylus* (as a valid subgenus of *Stibasoma*, error): *R. planiventris* (Wiedemann, 1828), *R. rubrum* (Thunberg, 1827), *R. venenatum* (Osten Sacken, 1886), and *R. viridiventris* (Macquart, 1838). Because of this continued discussion, here I revise the genus *Rhabdotylus* and redescribe three *Rhabdotylus* species, and propose one new synonym.

## Materials and methods

I examined the type specimens of all species of *Rhabdotylus*, which are deposited in the following institutions: The Natural History Museum, London, UK (BMNH); Museum für Naturkunde, Berlin, Germany (MFNB); Museum National d’Histoire Naturelle, Paris, France (MNHN); Fundação Instituto Oswaldo Cruz, Rio de Janeiro, Brazil (FIOC); and Uppsala University, Museum of Evolution, Zoology Section, Uppsala, Sweden (UUZM).

Terminology follows Cumming and Wood (Cumming and Wood 2017). Pinned specimens were examined under a stereomicroscope and whole habitus photographs were taken at the USNM using a GIGAmacro Magnify2 system, a Canon EOS D5 full-frame DSLR, a Canon MP-E 65 mm f2.8 macro-lens, using a twin-flash for lighting. The software HeliconFocus Pro (version 6.7.1) was used to stack individual RAW format images using Method C (pyramid), to add the scale, and to export the final image in Adobe DNG-format.

## Taxon treatments

### Rhabdotylus

Lutz, 1913

Rhabdotylus Lutz, 1909: 29 (1911: 34), *nomen nudum* (Lutz 1909, Lutz 1911).
Rhabdotylus **Rhabdotylus**[Bibr B5222493][Bibr B5222503][Bibr B5223119]*Tabanus planiventris*[Bibr B5222935][Bibr B5218420]*Tabanus rubrum*[Bibr B5222727][Bibr B5218566][Bibr B5218586]
Gymnochela Enderlein, 1925: 388 (Enderlein 1925). Type species: *Tabanus planiventris* Wiedemann, 1828 = *Tabanus rubrum* Thunberg, 1827.
Rhabdotylus

#### Diagnosis

Includes species with typically medium-sized bodies (13–15mm), greenish or yellow to brown; eyes bare; ocellar tubercle and ocelli indistinct; frontal callus as wide as frons, mid callus connected to frontal callus, both the same color as frons; subcallus bare; short robust antenna, basal flagellomere of antenna with long and curved dorsal spine; palpus shorter than proboscis; labellum totally sclerotized; wings hyaline, sometimes yellowish; legs slender, scarcely haired; abdomen with distal bands on tergites.

##### Species

*Rhabdotylus rubrum* (Thunberg, 1827), *R. venenatum* (Osten Sacken, 1886), *R. viridiventris* (Macquart, 1838).

### Rhabdotylus rubrum

(Thunberg, 1827)

Rhabdotylus rubrum ***Rhabdotylus rubrum****Tabanus*[Bibr B5222727][Bibr B5222674]*Stibasoma*Fairchild 1971*Stibasoma*[Bibr B5222539][Bibr B5218512]
Rhabdotylus planiventris (Wiedemann, 1828): 139 (*Tabanus*) (Wiedemann 1828), syn. nov.; Walker, 1854: 216 (*Tabanus*) (Walker 1854); Hunter, 1901: 143 (*Tabanus*) (Hunter 1901); Kertész, 1908: 269 (*Tabanus*) (Kertész 1908); Enderlein, 1925: 388 (*Gymnochela*) (Enderlein 1925); Kröber, 1932: 91 (*Gymnochela*) (Kröber 1932), 1934: 271 (*Amphichlorops*) (Kröber 1934); Pechuman, 1942: 55 (*Amphichlorops*) (Pechuman 1942); Carrera & Lane, 1945: 133 (Carrera and Lane 1945); Coscarón, 1967: 114 (Coscarón 1967); Fairchild, 1967a: 95 (Fairchild 1967a), 1971: 77 (*Stibasoma*) (Fairchild 1971), 1975: 262 (*Amphichlorops*) (Fairchild 1975); Moucha, 1976: 206 (*Stibasoma*) (Moucha 1976); Fairchild & Burger, 1994: 114 (*Stibasoma*) (Fairchild and Burger 1994); Turcatel et al., 2007: 276 (*Stibasoma*) (Turcatel et al. 2007), Coscarón & Papavero, 2009: 112 (*Stibasoma*) (Coscarón and Papavero 2009).
Pangonia cornuta Walker, 1837: 337 (Walker 1837); Kertész, 1908: 153 (Kertész 1908); Fairchild, 1956: 14 (Fairchild 1956) (synonym).
Tabanus lativentris Macquart, 1838: 153 (Macquart 1838); Blanchard, 1852: 393 (Blanchard 1852); Walker, 1854: 265 (Walker 1854); Philippi, 1865: 714 (Philippi 1865); Hunter, 1901: 141 (Hunter 1901); Kertész, 1908: 254 (Kertész 1908); Kröber, 1934: 274 (Kröber 1934); Fairchild, 1956: 20 (Fairchild 1956) (synonym).

#### Materials

- **Type status:** Holotype. **Occurrence:** individualCount: 1; sex: female; lifeStage: adult; **Taxon:** scientificName: Rhabdotylus rubrum; originalNameUsage: Tabanus rubrum; family: Tabanidae; genus: Rhabdotylus; specificEpithet: rubrum; scientificNameAuthorship: Thunberg, 1827; **Location:** locationRemarks: label transliteration: “Uppsala Univ. Zool. Mus. \ Thunbergsaml. nr. 22069 \ Tabanus ruber \ Amer. Merid. TYP” “Rhabdotylus \ nr. Viridivent- \ -ris M. \ C. B. Philip .66”; **Record Level:** institutionID: Uppsala University, Museum of Evolution, Zoology Section; institutionCode: UUZM; basisOfRecord: PreservedSpecimen
- **Type status:** Holotype. **Occurrence:** individualCount: 1; sex: female; lifeStage: adult; **Taxon:** scientificName: Rhabdotylus rubrum; originalNameUsage: Tabanus planiventris; family: Tabanidae; genus: Rhabdotylus; specificEpithet: rubrum; scientificNameAuthorship: Wiedemann, 1828; **Location:** country: Brazil; locationRemarks: label transliteration: “Brasil. V. Olf.” “98” “Type” “planiventris Wied.*” “Amphichlorops \ planiventris \ Wied. \ det. Kröber 1927” “Zool. Mus. Berlin”; **Record Level:** institutionID: Museum für Naturkunde; institutionCode: MFNB; basisOfRecord: PreservedSpecimen
- **Type status:** Other material. **Occurrence:** catalogNumber: UNSMENT01518377; recordedBy: Lauro Travassos Filho; individualCount: 1; sex: female; lifeStage: adult; **Taxon:** scientificName: Rhabdotylus rubrum; family: Tabanidae; genus: Rhabdotylus; specificEpithet: rubrum; **Location:** country: Brazil; countryCode: BR; stateProvince: Santa Catarina; municipality: Joinville; locationRemarks: label transliteration: "Sta. Catarina \ Joinville \ L. Trav. Fş" "Rhabdotylus \ planiventris (Wied.) \ Barretto det. 1948"; **Record Level:** institutionID: U. S. National Entomological Collection; institutionCode: USNM; basisOfRecord: PreservedSpecimen
- **Type status:** Other material. **Occurrence:** catalogNumber: USNMENT01518378; recordedBy: Raymond Corbett Shannon; individualCount: 1; sex: female; lifeStage: adult; **Taxon:** scientificName: Rhabdotylus rubrum; family: Tabanidae; genus: Rhabdotylus; specificEpithet: rubrum; **Location:** country: Brazil; countryCode: BR; stateProvince: Rio de Janeiro; municipality: Tinguá; locationRemarks: label transliteration: "Tinguá \ R. Janeiro \ Brasil" "Dezembro \ 1950" "RCShannon Collection" "Serviço Febre \ Amarela \ M. E. S., Bras."; **Record Level:** institutionID: U. S. National Entomological Collection; institutionCode: USNM; basisOfRecord: PreservedSpecimen
- **Type status:** Other material. **Occurrence:** catalogNumber: USNMENT01518379; recordedBy: F. Schade; individualCount: 1; sex: female; lifeStage: adult; **Taxon:** scientificName: Rhabdotylus rubrum; family: Tabanidae; genus: Rhabdotylus; specificEpithet: rubrum; **Location:** country: Brazil; countryCode: BR; locationRemarks: label transliteration: "C. Brazil \ Dec. 1935 \ F. Schade" "ALMelander \ Collection" "St. (Rhabdotylus) \ planiventris Wied. \ det. Fairchild 1963"; **Record Level:** institutionID: U. S. National Entomological Collection; institutionCode: USNM; basisOfRecord: PreservedSpecimen
- **Type status:** Other material. **Occurrence:** catalogNumber: USNMENT01518380; recordedBy: Raymond Corbett Shannon; individualCount: 1; sex: female; lifeStage: adult; **Taxon:** scientificName: Rhabdotylus rubrum; family: Tabanidae; genus: Rhabdotylus; specificEpithet: rubrum; **Location:** country: Brazil; countryCode: BR; stateProvince: Rio de Janeiro; municipality: Itatiaia; locationRemarks: label transliteration: "Alt. 200M. \ Itatiaia \ R. Janeiro" "Fevereiro \ 1941" "RCShannon Collection"; **Record Level:** institutionID: U. S. National Entomological Collection; institutionCode: USNM; basisOfRecord: PreservedSpecimen
- **Type status:** Other material. **Occurrence:** catalogNumber: USNMENT01518381; recordedBy: Raymond Corbett Shannon; individualCount: 1; sex: female; lifeStage: adult; **Taxon:** scientificName: Rhabdotylus rubrum; family: Tabanidae; genus: Rhabdotylus; specificEpithet: rubrum; **Location:** country: Brazil; countryCode: BR; stateProvince: Rio de Janeiro; municipality: Mangaratiba; locationRemarks: label transliteration: "Mangaratiba \ R d Janeiro X \ Brazil XI'38" "Yel Fev Serv \ MES Brazil \ RCShannon"; **Record Level:** institutionID: U. S. National Entomological Collection; institutionCode: USNM; basisOfRecord: PreservedSpecimen

#### Description

Female (holotype). *Head.* Frons narrow, light brown, with sparse black hairs and silver pollinosity along the margins of the eyes. Frontal Index: 4.10. Divergence Index: 0.92. Subcallus light brown, with dense white pollinosity. Scape and pedicel light brown, with black and some white hairs mixed and white pollinosity. Flagellum orange (missing on holotype). Clypeus light brown, with dense white pollinosity and sparse white hairs. Gena and postgena light brown, with dense white pollinosity and dense white hairs. Palpus yellow to light brown, with sparse white pollinosity and mixed white and black hairs.

*Thorax.* Scutum and scutellum light brown with sparse white to yellow and black hairs mixed. Postpronotal lobe light brown with white pollinosity, white and black hairs mixed. Notopleuron light brown with white pollinosity, white to yellow and black hairs. Proepisternum and proepimerum light brown with white pollinosity and white hairs. Anepisternum light brown with white pollinosity, white to yellow and black hairs mixed. Katepisternum light brown with white pollinosity and white to yellow hairs. Anepimeron light brown with white pollinosity, with white to yellow hairs. Katepimeron and katatergite light brown with white pollinosity and white hairs.

Coxa light brown with white pollinosity with white hairs, and some black hairs distally. Femur 1 light brown with black hairs. Femur 2 light brown with black hairs and a tuft of white hairs proximally on posterodorsal side. Femur 3 light brown with black and white hairs mixed. Tibiae 1 and 2 yellow to light brown with white hairs proximally, and brown with black hairs distally. Tibia 3 brown with black hairs. Tarsus brown with black hairs. Wing hyaline. Pterostigma yellow. Venation light brown. Halter yellow, with white apex.

*Abdomen.* Abdomen predominantly light brown with sparse black hairs and white hairs laterally. Tergite 1 with a faintly lighter band distally and white hairs in the middle of the distal margin. Tergites 2–4 with a faintly lighter band distally. Sternites 1–3 yellow to light brown with a narrow white band distally and white hairs. Remaining sternites light brown with a narrow white band distally, with black hairs proximally and laterally and white hairs distally.

*Measurements*: Body length: 15mm. Wing length: 12.5mm.

Figs 1, 2, 3

#### Distribution

Brazil (Rio de Janeiro, São Paulo, Paraná, Santa Catarina), Argentina.

### Rhabdotylus venenatum

(Osten Sacken, 1886)

Rhabdotylus venenatum ***Rhabdotylus venenatum****Tabanus*Osten Sacken 1886*Tabanus*Aldrich 1905*Tabanus*Kertész 1908*Gymnochela*Kröber 1932*Ampichlorops*Kröber 1934*Amphichlorops*[Bibr B5222600][Bibr B5222717][Bibr B5222717][Bibr B5222655]*Stibasoma*Fairchild 1961b*Stibasoma*[Bibr B5218660][Bibr B5222313]*Stibasoma*Hogue and Fairchild 1974*Amphichlorops*Fairchild 1975*Stibasomavenatum*Moucha 1976*Stibasoma*[Bibr B5222954][Bibr B5222333]*Stibasoma*Chainey 1990*Stibasoma*Fairchild and Burger 1994*Stibasoma*Coscarón and Papavero 2009

#### Materials

- **Type status:** Lectotype. **Occurrence:** catalogNumber: NHMUK012805335; recordedBy: F. . Godman, O. Salvin; individualCount: 1; sex: female; lifeStage: adult; **Taxon:** scientificName: Rhabdotylus venenatum; originalNameUsage: Tabanus venenatus; family: Tabanidae; genus: Rhabdotylus; specificEpithet: venenatum; scientificNameAuthorship: Osten Sacken, 1886; **Location:** country: Panama; countryCode: PA; stateProvince: Chiriqui; locationRemarks: label transliteration: “LECTO- \ TYPE” “Co- \ type” “Tabanus \ venenatus \ O. S.” “V. de Chiriqui, \ 2-2000 ft. \ Champion.” “Central America. \ Pres. By \ F. D. Godman, \ O. Salvin. \ 1904–85” “LECTOTYPE \ Stibasoma \ venenata \ Osten Sacken \ det. \ C. B. Philip 53” “BMNH(E) # \ 253483”; **Record Level:** institutionID: The Natural History Museum, London; institutionCode: BNHM; basisOfRecord: PreservedSpecimen
- **Type status:** Paralectotype. **Occurrence:** catalogNumber: NHMUK012805336; recordedBy: F. . Godman, O. Salvin; individualCount: 1; sex: female; lifeStage: adult; **Taxon:** scientificName: Rhabdotylus venenatum; originalNameUsage: Tabanus venenatus; family: Tabanidae; genus: Rhabdotylus; specificEpithet: venenatum; scientificNameAuthorship: Osten Sacken, 1886; **Location:** country: Guatemala; countryCode: GT; locality: Sinanjá; locationRemarks: label transliteration: “PARA- \ LECTO- \ TYPE” “Co- \ type” “Tabanus \ venenatus \ O. S.” “Sinanja, \ Vera Paz. \ Champion.” “Central America. \ Pres. By \ F. D. Godman, \ O. Salvin. 1904–85.” “BMNH(E) # \ 253484”; **Record Level:** institutionID: The Natural History Museum, London; institutionCode: BNHM; basisOfRecord: PreservedSpecimen
- **Type status:** Other material. **Occurrence:** catalogNumber: USNMENT01518382; recordedBy: Andrea Langley, Jeffrey Cohen; individualCount: 1; sex: female; lifeStage: adult; **Taxon:** scientificName: Rhabdotylus venenatum; family: Tabanidae; genus: Rhabdotylus; specificEpithet: venenatum; **Location:** country: Ecuador; countryCode: EC; stateProvince: Cotopaxi; locality: Latacunga; locationRemarks: label transliteration: "ECUADOR Cotopaxi \ Latacunga, 117 Km W \ 1 July 1975 3000'\ Andrea Langley \ Jeffrey Cohen" "Ecuador - Peace Corps \ Smithsonian Institution \ Aquatic Insect Survey" "Stibasoma \ (Rhabdotylus) \ venenata O. S. \ Det. 1976 \ G. B. Fairchild"; **Record Level:** institutionID: U. S. National Entomological Collection; institutionCode: USNM; basisOfRecord: PreservedSpecimen
- **Type status:** Other material. **Occurrence:** catalogNumber: USNMENT01518383; recordedBy: Pab. Schild, A. L. Melander; individualCount: 1; sex: female; lifeStage: adult; **Taxon:** scientificName: Rhabdotylus venenatum; family: Tabanidae; genus: Rhabdotylus; specificEpithet: venenatum; **Location:** country: Costa Rica; countryCode: CR; locality: La Suiza; locationRemarks: label transliteration: "I 01" "Costa Rica \ La Suiza '23 \ Pab. Schild" "ALMelander \ Collection \ 1961" "St. (Rhabdotylus) \ venenata O. S. \ det. Fairchild 1963"; **Record Level:** institutionID: U. S. National Entomological Collection; institutionCode: USNM; basisOfRecord: PreservedSpecimen
- **Type status:** Other material. **Occurrence:** catalogNumber: USNMENT01518384; recordedBy: R. J. Anduze; individualCount: 1; sex: female; lifeStage: adult; **Taxon:** scientificName: Rhabdotylus venenatum; family: Tabanidae; genus: Rhabdotylus; specificEpithet: venenatum; **Location:** country: Venezuela; countryCode: VE; stateProvince: Miranda; locality: San Carlos; locationRemarks: label transliteration: "San Carlos \ Estado Miranda \ Venez. 17.VIII.42 \ 1460 mts \ R. J. Anduze" "Stibasoma \ (Rhabdotylus) \ venenatus O.S. \ det. Fairchild 1955"; **Record Level:** institutionID: U. S. National Entomological Collection; institutionCode: USNM; basisOfRecord: PreservedSpecimen

#### Description

*Female* (holotype). *Head.* Frons narrow, light brown, with sparse black hairs and silver pollinosity along the margins of the eyes. Frontal Index: 3.65. Divergence Index: 1. Subcallus yellow, with dense yellow pollinosity. Scape and pedicel yellow to light brown, with black hairs and some yellow hairs mixed and some yellow pollinosity. Flagellum orange with short white hairs, apical flagellomeres missing. Clypeus yellow with dense yellow pollinosity and yellow hairs. Gena and postgena yellow with dense yellow pollinosity and yellow hairs. Palpus yellow with yellow hairs.

*Thorax.* Scutum light brown with sparse black hairs, scutellum yellow to green with sparse black hairs and white pollinosity. Postpronotal lobe and notopleuron yellow to light brown, with white pollinosity and black hairs. Proepisternum and proepimerum yellow with white pollinosity and yellow hairs. Anepisternum, katepisternum, anepimeron, katepimeron, and katatergite yellow with white pollinosity and yellow hairs. Coxa yellow, with white pollinosity and yellow hairs. Femur yellow with yellow hairs and slightly darker with black hairs distally. Tibiae 1 and 2 yellow with white hairs in proximal half, distal half brown with dark brown to black hairs. Tibia 3 brown with dark brown to black hairs. Tarsus brown with dark brown to black hairs. Pterostigma yellow. Venation brown. Halter yellow, with white apex.

*Abdomen.* Abdomen predominantly yellow to green with sparse black hairs, and white hairs laterally on tergites 1–5. Tergites 1–3 yellow to green with a lighter band distally and sparse black hairs. Remaining tergites slightly darker and with more black hairs distally. Sternites yellow with a thin lighter band distally, and white to yellow hairs.

*Measurements*: Body length: 16mm. Wing length: 15mm.

Figs 4, 5, 6

#### Distribution

Guatemala, Costa Rica, Panama, Colombia, Venezuela, Ecuador, Peru.

### Rhabdotylus viridiventris

(Macquart, 1838)

Rhabdotylus viridiventris ***Rhabdotylus viridiventris****Tabanus*Macquart 1838*Tabanus*Walker 1854*Tabanus*Hunter 1901*Tabanus*Kertész 1908*Gymnochela*Kröber 1932*Amphichlorops*[Bibr B5222415][Bibr B5218454][Bibr B5218576]*Stibasoma*Fairchild 1971*Stibasoma*[Bibr B5222539][Bibr B5222323][Bibr B5222737][Bibr B5218512]
Dicladocera sulphurea Kröber, 1931: 408 (Kröber 1931). Kröber, 1934: 269 (Kröber 1934), 1940: 83 (Kröber 1940); Pechuman, 1942: 55 (Pechuman 1942); Fairchild, 1967b: 344 (Fairchild 1967b) (synonym).

#### Materials

- **Type status:** Holotype. **Occurrence:** individualCount: 1; sex: female; lifeStage: adult; **Taxon:** scientificName: Rhabdotylus viridiventris; originalNameUsage: Tabanus viridiventris; family: Tabanidae; genus: Rhabdotylus; specificEpithet: viridiventris; scientificNameAuthorship: Macquart, 1838; **Location:** country: Brazil; countryCode: BR; stateProvince: Rio de Janeiro; municipality: Rio de Janeiro; locationRemarks: label transliteration: “Tabanus \ viridiventris” “Rio-janei. \ St. hilaire” “IOC” “HOLOTYPE” “MNHN, Paris \ ED7586”; **Record Level:** institutionID: Museum National d'Histoire Naturelle; institutionCode: MNHN; basisOfRecord: PreservedSpecimen
- **Type status:** Other material. **Occurrence:** recordedBy: R. Benoist; individualCount: 1; sex: female; lifeStage: adult; **Taxon:** scientificName: Rhabdotylus viridiventris; family: Tabanidae; genus: Rhabdotylus; specificEpithet: viridiventris; **Location:** country: Ecuador; countryCode: EC; municipality: Quito; locationRemarks: label transliteration: "Équateur \ Quito" "Muséum Paris" "Équateur \ R. Benoist 1930" "Stibasoma (Rhabdotylus) \ viridiventris Macq. \ Det. Fairchild 1965"; **Record Level:** institutionID: Museum National d'Histoire Naturelle; institutionCode: MNHN; basisOfRecord: PreservedSpecimen
- **Type status:** Other material. **Occurrence:** individualCount: 1; sex: female; lifeStage: adult; **Taxon:** scientificName: Rhabdotylus viridiventris; family: Tabanidae; genus: Rhabdotylus; specificEpithet: viridiventris; **Location:** country: Brazil; countryCode: BR; locationRemarks: label transliteration: "R. Bandeirante \ 7.7.37" "Rhabdotylus (Lutz) \ viridiventris \ Macquart \ Leg. B. Lutz \ (nymphas)" "N. T636" "Inst. O. Cruz" "Coleçăo A. Lutz"; **Record Level:** institutionID: Fundaçăo Instituto Oswaldo Cruz; institutionCode: FIOC; basisOfRecord: PreservedSpecimen

#### Description

*Female* (holotype). *Head.* Frons narrow, light brown, with sparse black hairs and silver pollinosity along the margins of the eyes. Frontal Index: 4.56. Divergence Index: 1. Subcallus light brown with dense white pollinosity. Scape and pedicel yellow to light brown with black hairs. Flagellum orange. Clypeus light brown with dense white pollinosity and white hairs. Gena and postgena light brown with dense white pollinosity and white hairs. Palpus yellow with black hairs.

*Thorax.* Scutum light brown with sparse white pollinosity and sparse black hairs, and a tuft of white and black hairs on the supra-alar area; scutellum light brown to yellow with sparse white pollinosity and a few white and black hairs. Postpronotal lobe and notopleuron light brown, with white pollinosity and sparse black hairs. Proepisternum and proepimerum light brown, with white pollinosity and white hairs. Anepisternum light brown with white pollinosity and white hairs anteriorly and a tuft of black hairs posterodorsally, near the wing. Katepisternum, anepimeron, katepimeron, and katatergite light brown with white pollinosity and white hairs. Coxa 1 yellow with white pollinosity and white hairs on proximal two-thirds, and light brown with white pollinosity and some white hairs on distal third. Coxae 2 and 3 yellow to light brown, with white pollinosity and white and some black hairs mixed. Femur yellow to light brown with black hairs. Tibiae 1 and 2 light brown to yellow with white hairs proximally, and light brown with black hairs distally. Tibia 3 light brown with black hair. Tarsus light brown with black hairs. Pterostigma yellow. Venation brown. Halter yellow with white apex.

*Abdomen.* Abdomen predominantly light brown. Tergites 1–3 light brown with black hairs, with a faintly lighter band distally and some yellow to white hairs. Remaining tergites light brown with black hairs. Sternites 1–3 yellow to light brown with a lighter band distally and yellow to white hairs. Remaining sternites light brown with black hairs.

*Measurements*: Body length: 13.5mm. Wing length: 12mm.

Figs 7, 8

#### Distribution

Venezuela; Ecuador; Brazil (Minas Gerais, Rio de Janeiro, São Paulo, Paraná, Santa Catarina).

## Identification Keys

### Key to females of *Rhabdotylus*

**Table:** 

1	Anepisternum with a tuft of black hairs	***Rhabdotylus viridiventris***
–	Anepisternum without a tuft of black hairs	2
2	Scutellum and abdomen greenish; palpus yellow with yellow hairs (only a few black hairs distally)	***Rhabdotylus venenatum***
–	Scutellum and abdomen light brown to yellow; palpus yellow with black and white hairs mixed	***Rhabdotylus rubrum***

## Discussion

*Rhabdotylus planiventris* (Fig. 2) is here established as a junior synonym of *R. rubrum*, and this species may present some color variation on the abdomen. *Rhabdotylus rubrum* and *R. venenatum* display differences in color and have distinct geographic distributions, and the apparent absence of intermediate forms suggests that these are indeed separate species. New collecting efforts are needed to increase the representation of this genus in entomological collections and to provide fresh samples for phylogenetic studies, which are necessary to infer the placement of *Rhabdotylus* in relation to *Stibasoma* and other Diachlorini genera.

## Supplementary Material

XML Treatment for Rhabdotylus

XML Treatment for Rhabdotylus rubrum

XML Treatment for Rhabdotylus venenatum

XML Treatment for Rhabdotylus viridiventris

## Figures and Tables

**Figure 1a.**
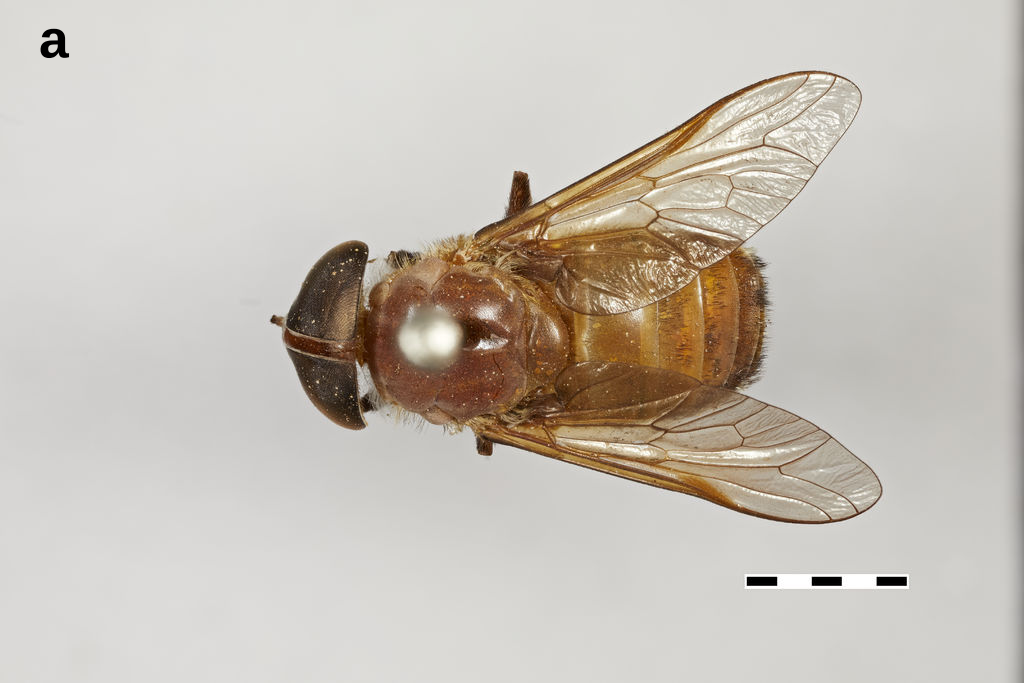
dorsal view

**Figure 1b.**
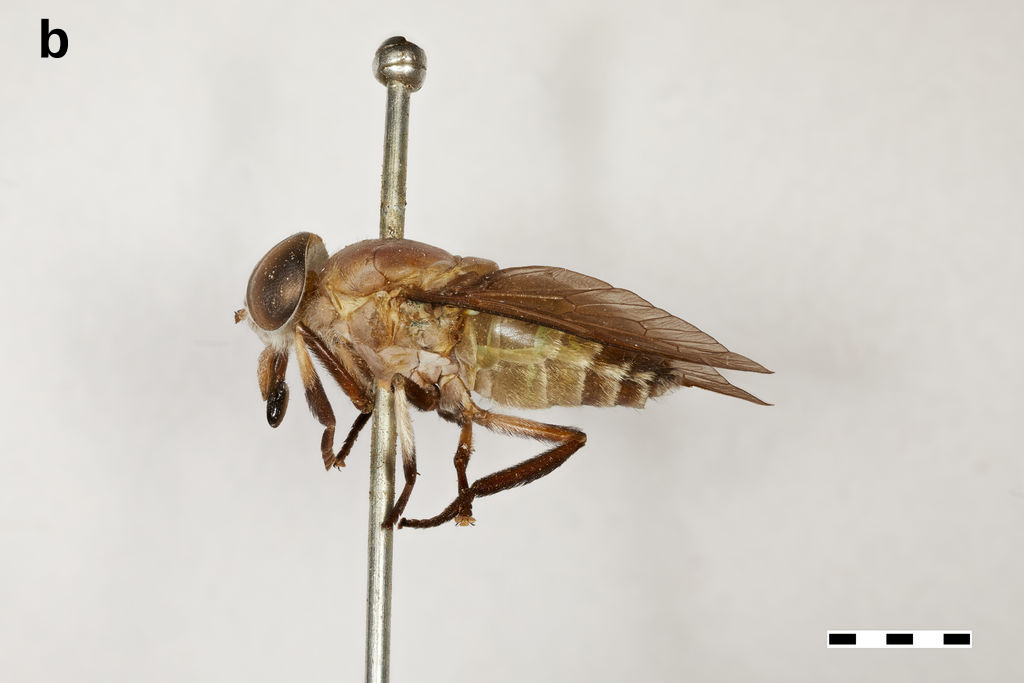
lateral view

**Figure 1c.**
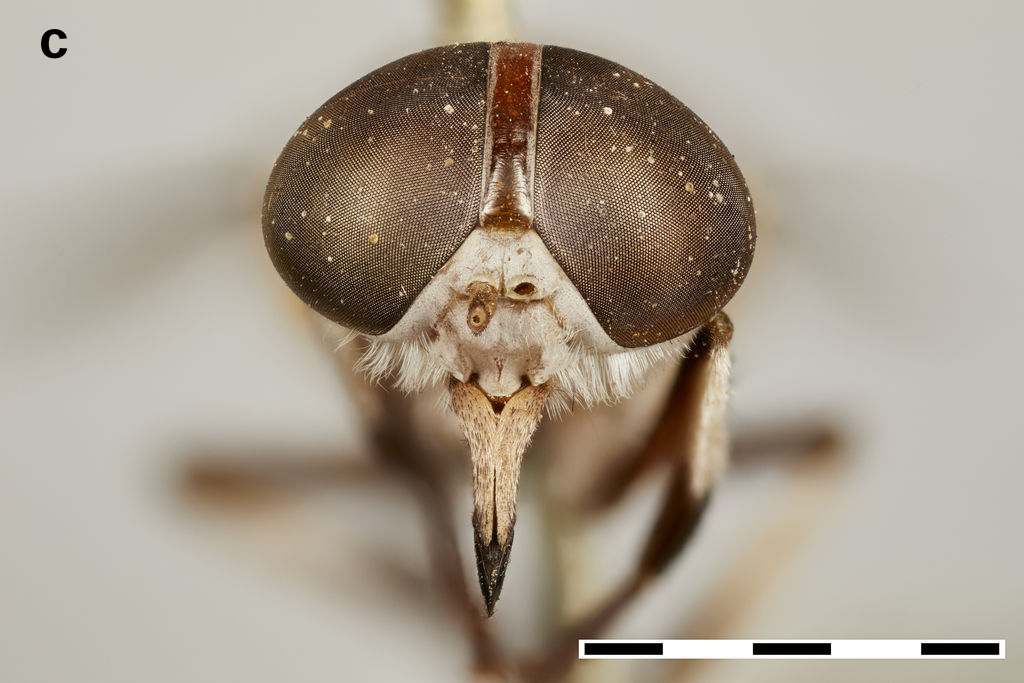
frontal view

**Figure 1d.**
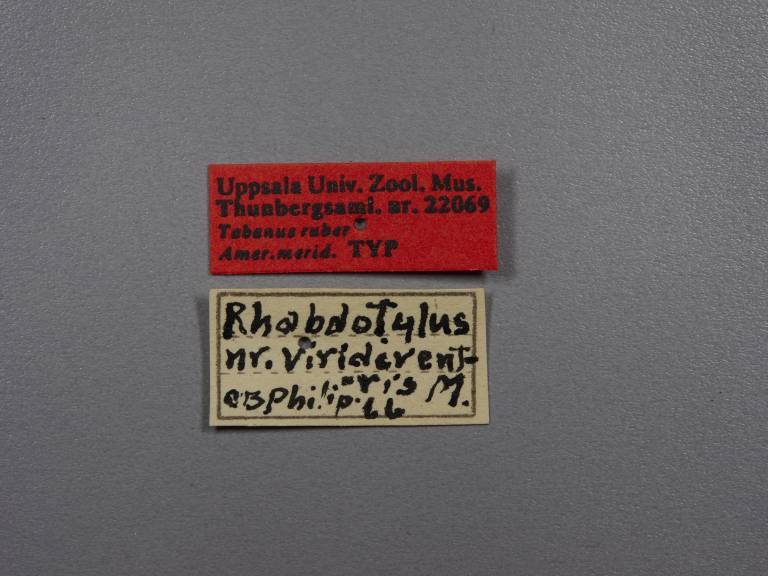
labels

**Figure 2a.**
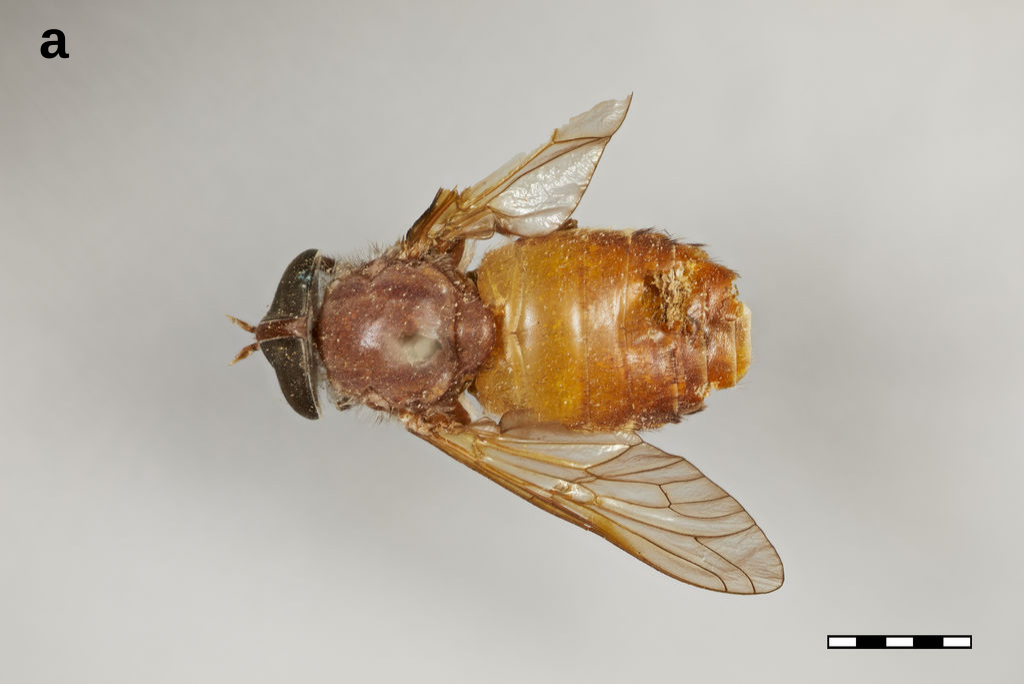
dorsal view

**Figure 2b.**
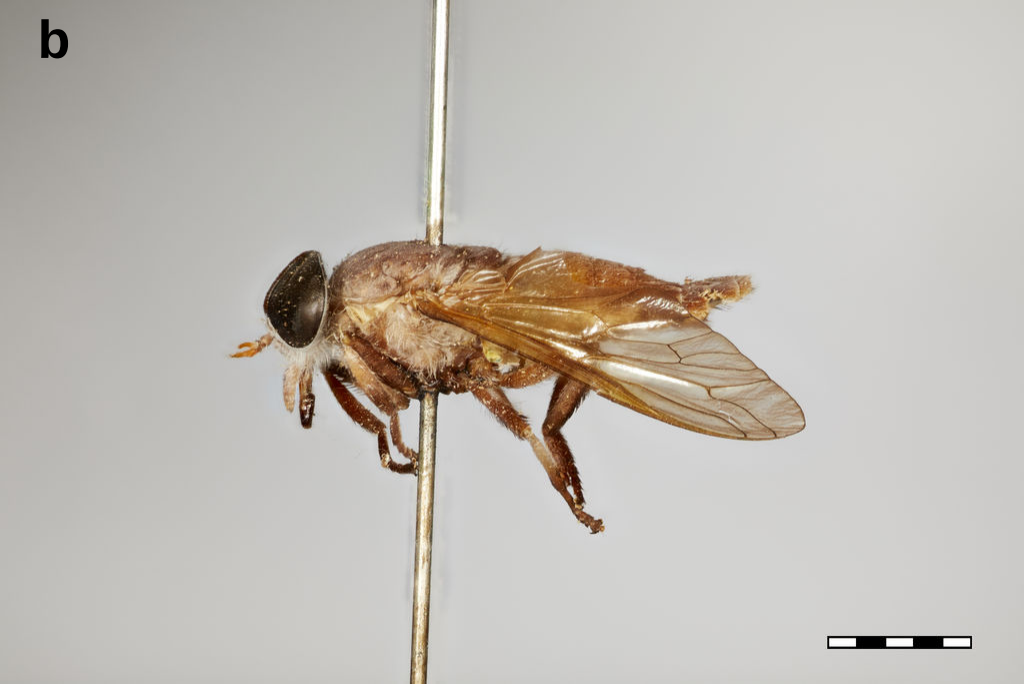
lateral view

**Figure 2c.**
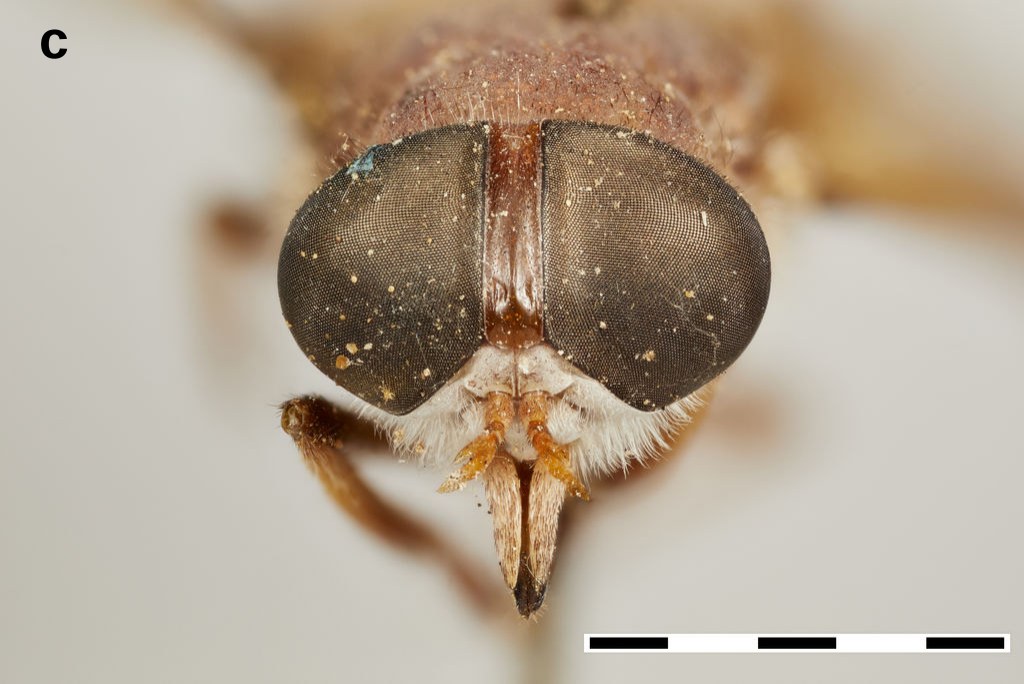
frontal view

**Figure 2d.**
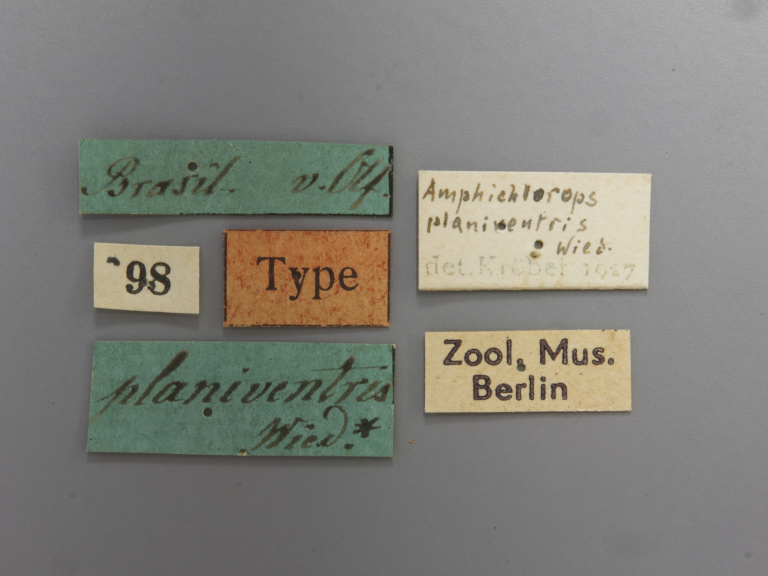
labels

**Figure 3.**
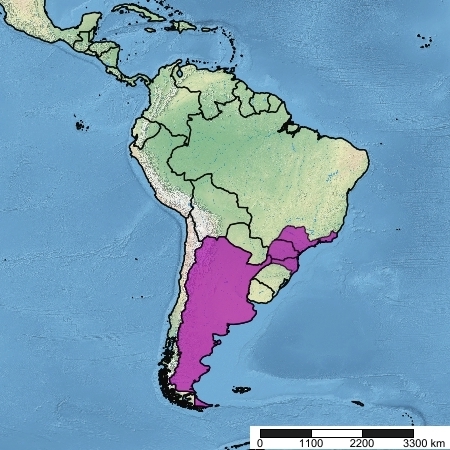
Known geographic range of *Rhabdotylus rubrum* (Thunberg, 1827)

**Figure 4a.**
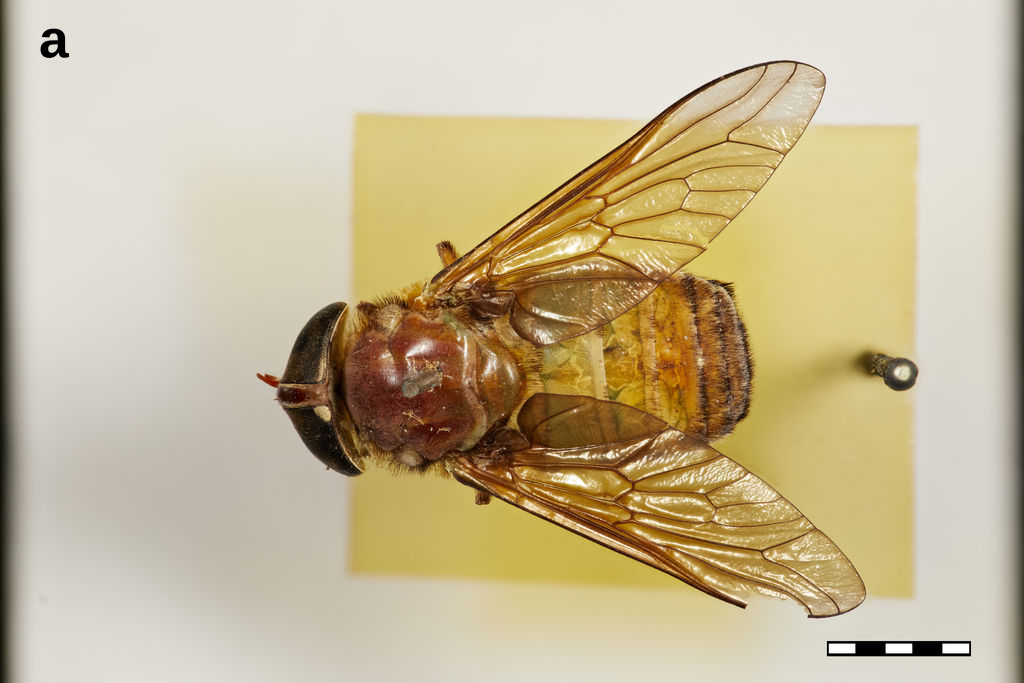
dorsal view

**Figure 4b.**
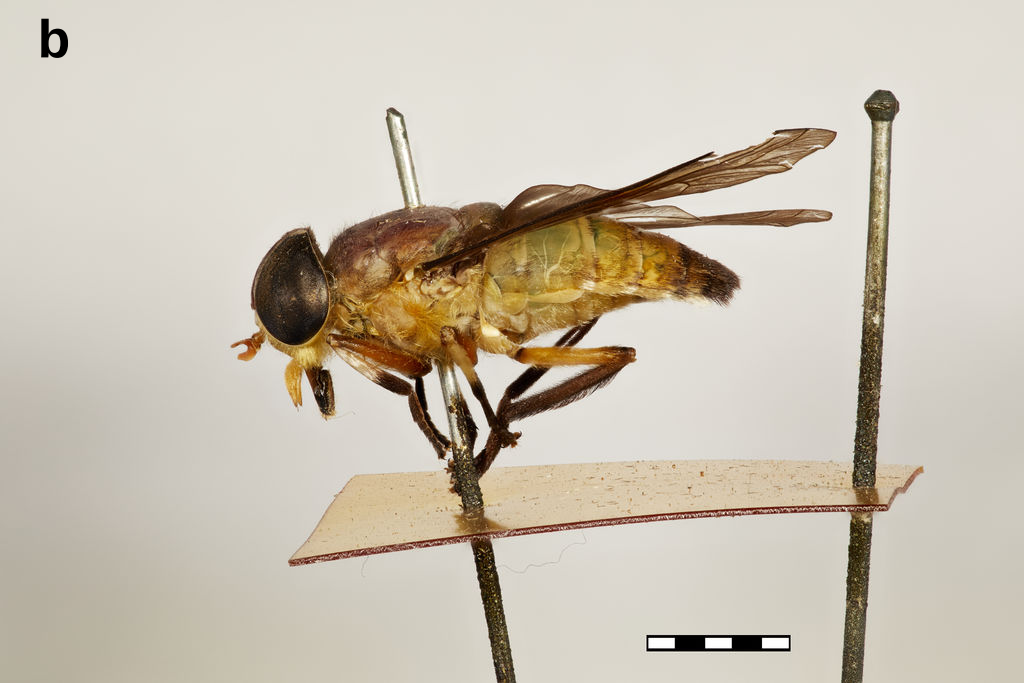
lateral view

**Figure 4c.**
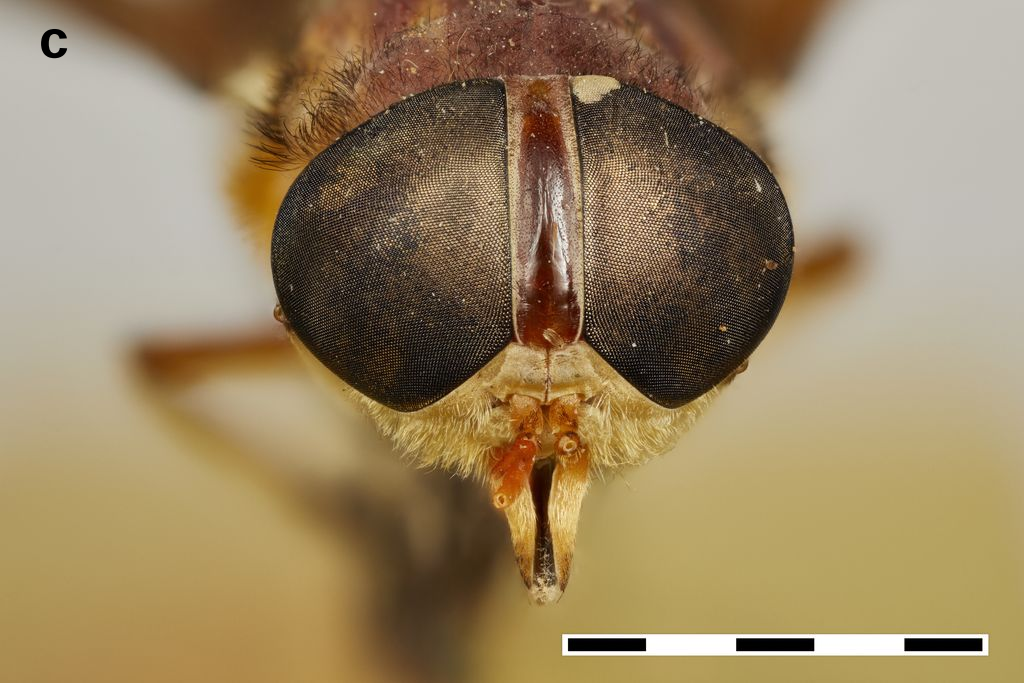
frontal view

**Figure 4d.**
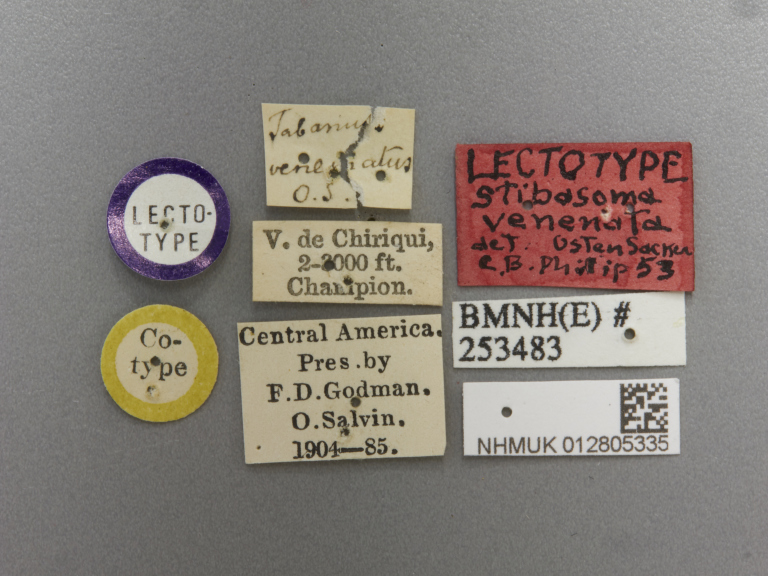
labels

**Figure 5a.**
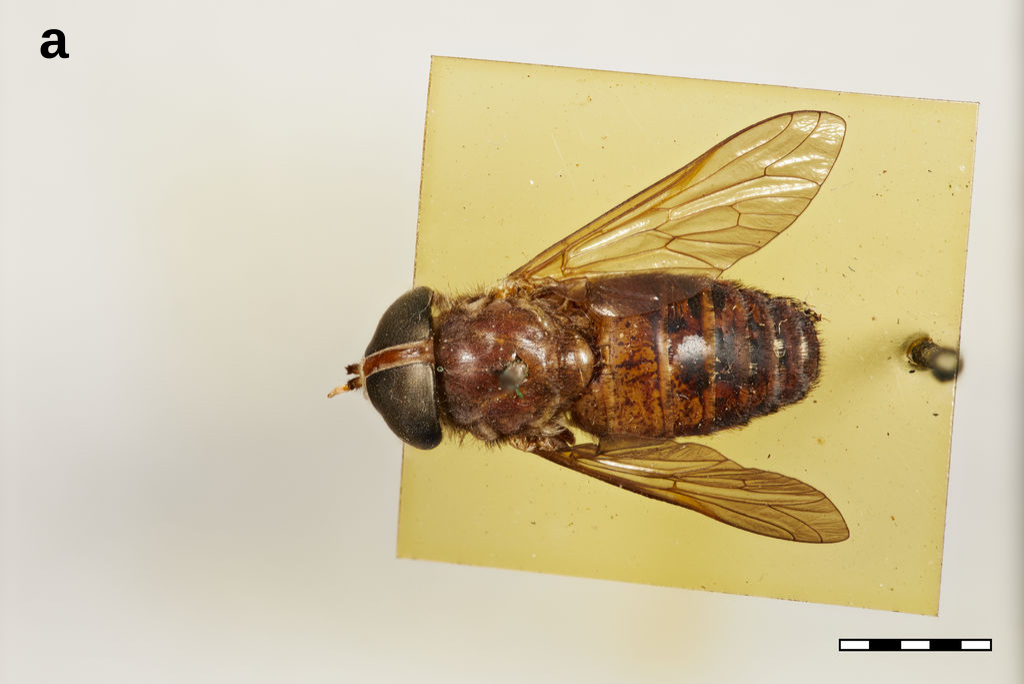
dorsal view

**Figure 5b.**
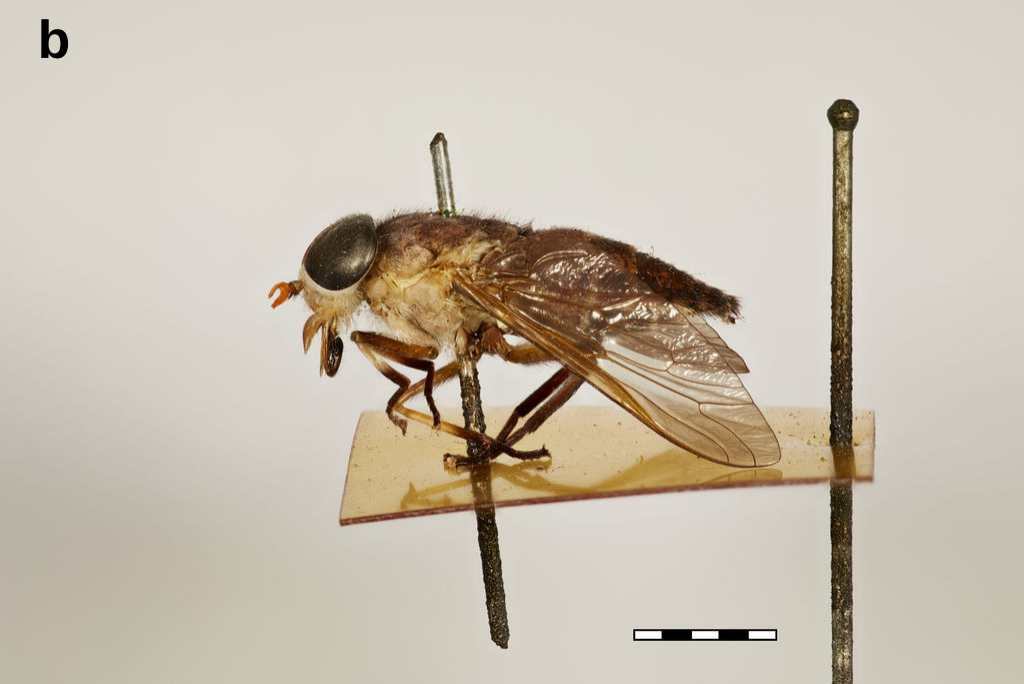
lateral view

**Figure 5c.**
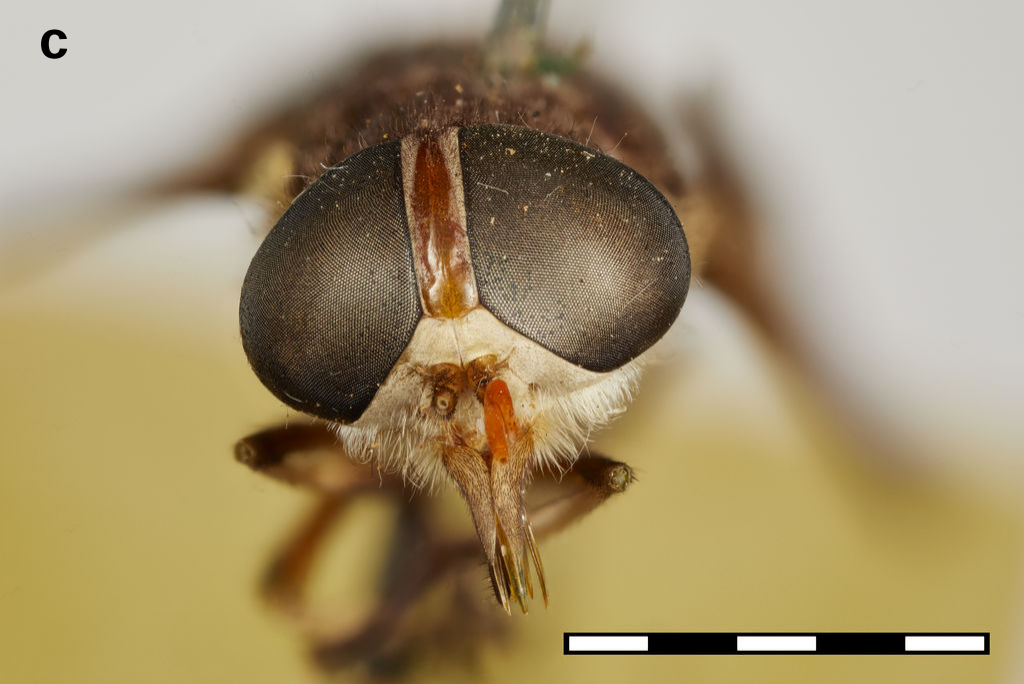
frontal view

**Figure 5d.**
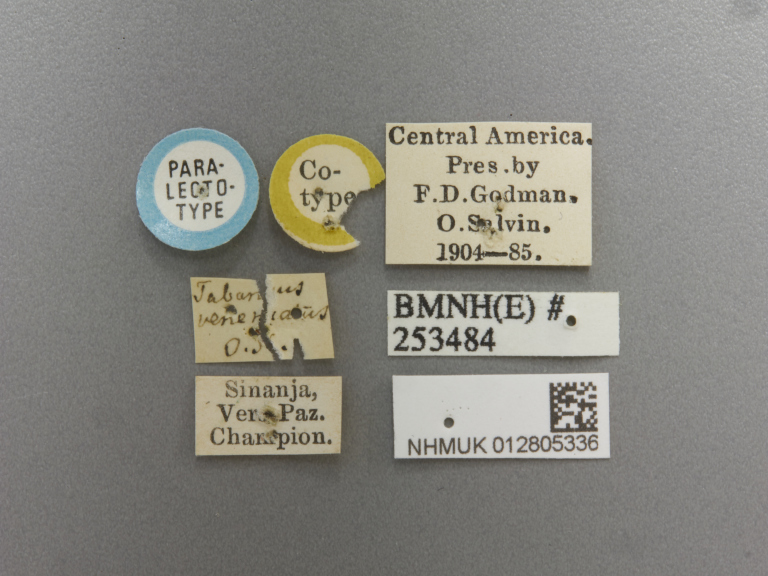
labels

**Figure 6.**
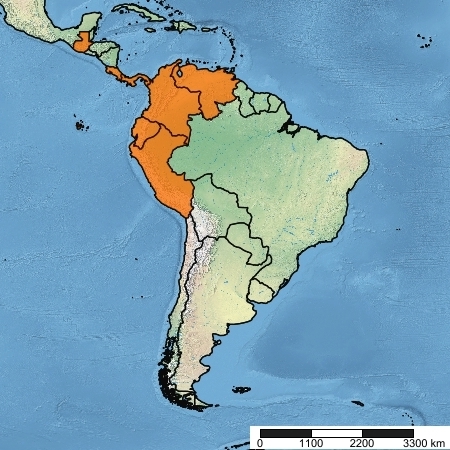
Known geographic range of *Rhabdotylus venenatum* (Osten Sacken, 1886).

**Figure 7a.**
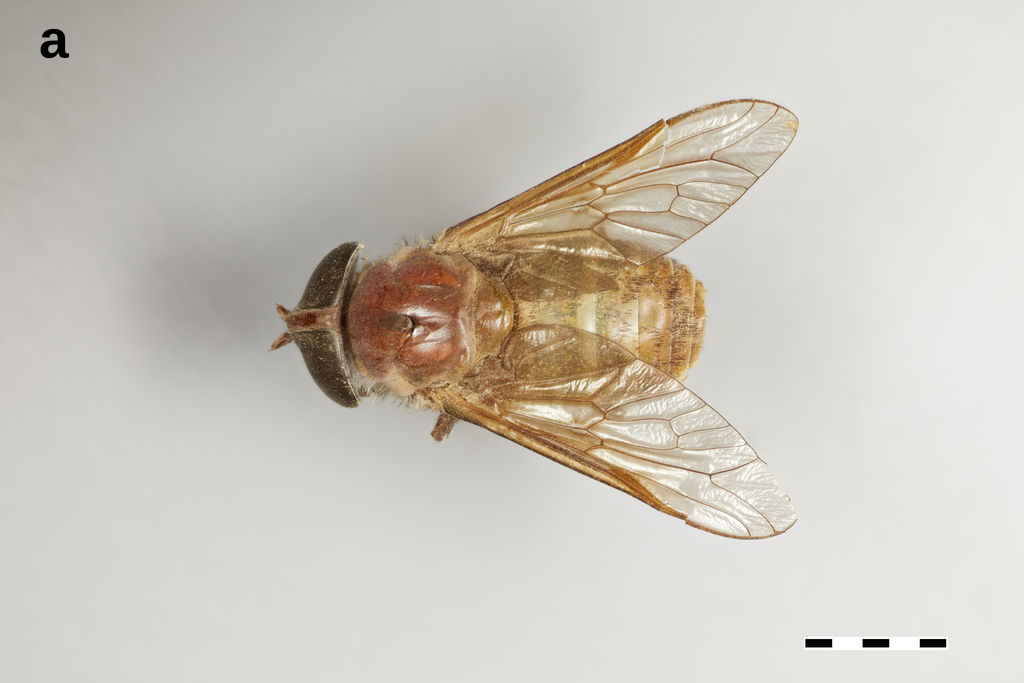
dorsal view

**Figure 7b.**
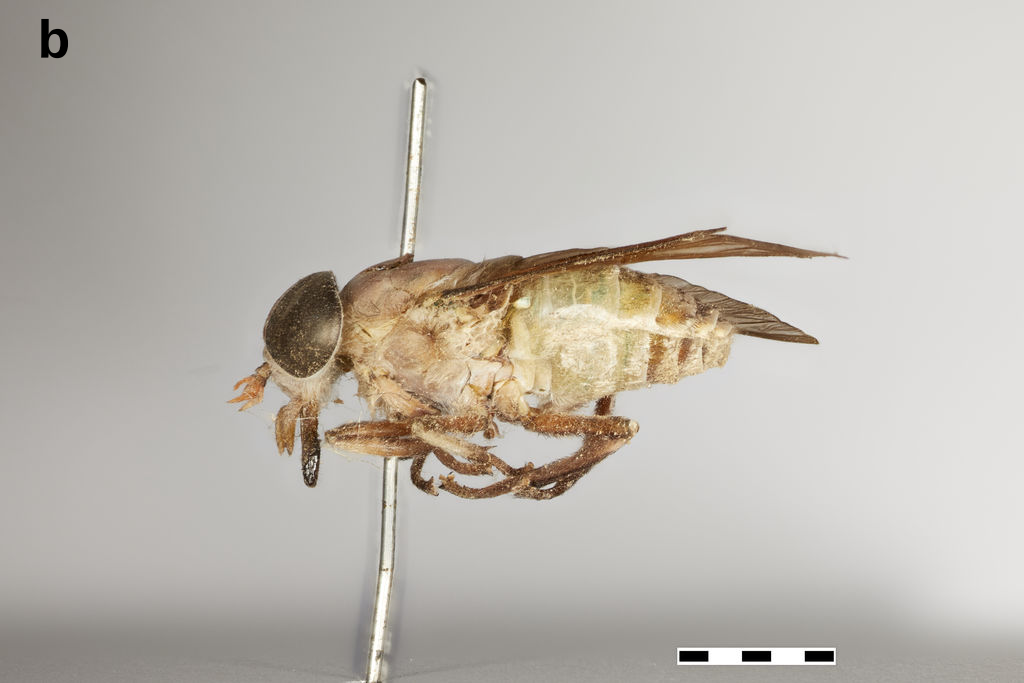
lateral view

**Figure 7c.**
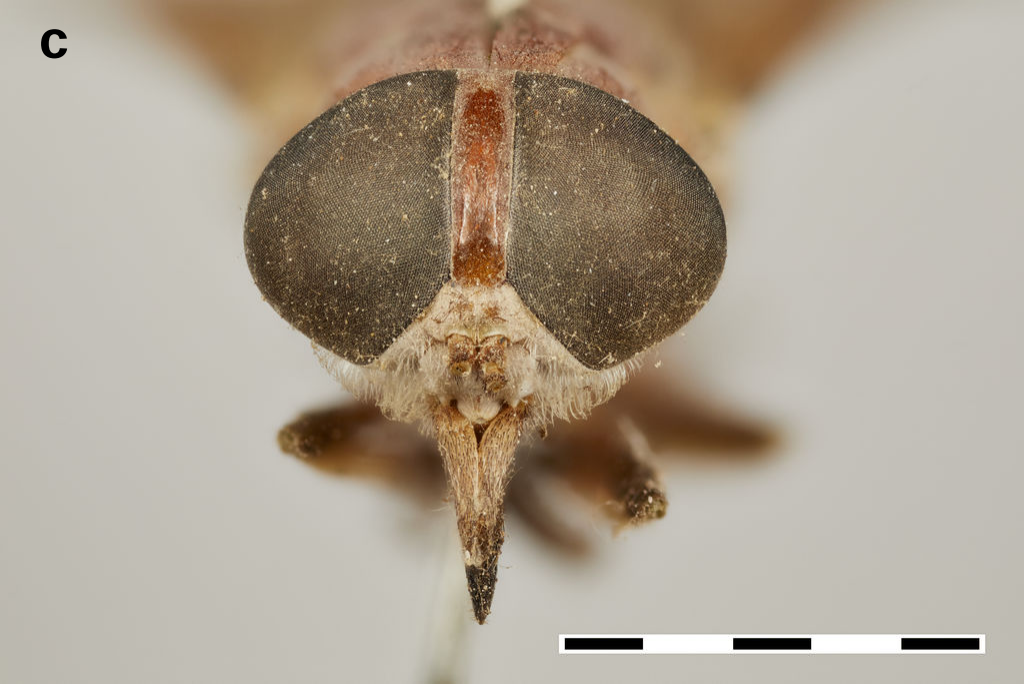
frontal view

**Figure 7d.**
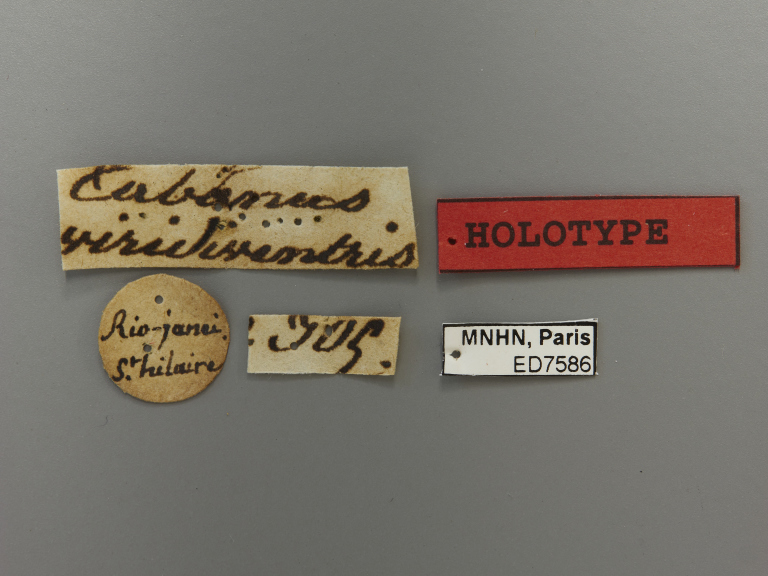
labels

**Figure 8.**
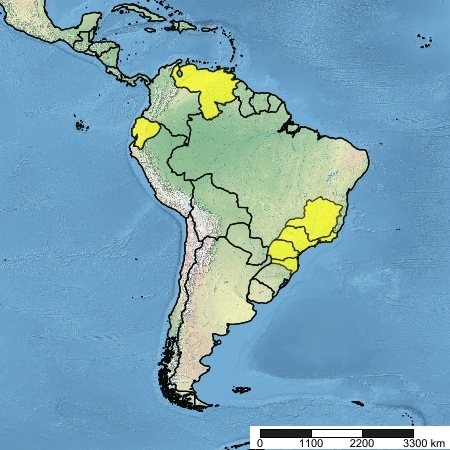
Known geographic range of *Rhabdotylus viridiventris* (Macquart, 1838).
